# ANA-Negative Presentation of SLE in Man with Severe Autoimmune Neutropenia

**DOI:** 10.1155/2016/6853936

**Published:** 2016-12-19

**Authors:** Melissa Zhao

**Affiliations:** University of California San Diego School of Medicine, La Jolla, CA, USA

## Abstract

*Background*. Systemic lupus erythematosus (SLE) is a chronic, inflammatory, connective tissue disease that commonly affects the joints and a variety of organs due to an overactivation of the body's immune system. There is wide heterogeneity in presentation of SLE patients, including lung, central nervous system, skin, kidney, and hematologic manifestations.* Case Presentation*. We report a case of atypical manifestation of SLE in a 53-year-old man who presented with neutropenic fever. Physical findings of interest included oral ulcers on the lower lip, a malar-like rash across the bridge of the nose, and a discoid-like rash on extensor surfaces of the elbows and knees. Labs include ANC <100, weakly positive anti-dsDNA, negative ANA, ferritin 1237 ng/mL, low C3/C4, and positive direct Coombs' test. A thorough workup for infection and hematologic malignancy was negative. Two days after initiation of therapy with 25 mg IV solumedrol twice a day, the patient's daily fevers resolved. ANC drastically improved to 2000 after two weeks of steroid treatment. He was later found to have a high titer of anti-neutrophil antibodies.* Discussion*. Autoimmune leukopenia is a common presentation in SLE, occurring in 50–60% of patients. Severe autoimmune neutropenia is uncommon and may correlate with high anti-neutrophil antibody activity despite a negative ANA. As neutropenia is usually mild, there are currently no guidelines for therapy. For our patient, we started him on low dose IV solumedrol and found that he responded drastically to treatment. Given strongly positive nonspecific anti-neutrophil antibodies in the setting of a negative ANA noted in our patient, it is likely that there are other currently unknown antibodies associated with SLE which may correlate strongly with autoimmune neutropenia.

## 1. Background

SLE is a chronic, inflammatory, connective tissue disease caused by an overactivation of the immune system. It has the potential to manifest in every organ of the human body, including the skin, central nervous system, lungs, kidneys, muscle, and blood. Of the hematologic manifestations, autoimmune leukopenia occurs in 50–60% of patients, involving lymphocytes or neutrophils, or a combination of both. Though most patients present with mild decrease of WBCs, less than 5% of patients present with severe autoimmune neutropenia with WBC count <500 cells/uL [1].

Due to the heterogeneity in presentation of SLE patients, no SLE diagnostic criteria have been created to date. The diagnosis of SLE is therefore largely based on clinical judgment after ruling out alternate diagnoses, with certain presentations more sensitive and predictive of disease than others, such as the presence of ANA and anti-Smith antibodies [2]. SLE can be detected in the serum with a variety of laboratory markers, such as ANA, anti-dsDNA, anti-Smith, anti-SSA, and anti-SSB [2], though these markers range in sensitivity of 26–57% and specificity of 95.9–98.6% [3]. SLE is also associated with increased serum concentrations of ferritin, decreased iron, and decreased C3/C4 levels [4]. Though diagnostic criteria for SLE currently do not exist due to the variety of disease presentation, two classification criteria, 1997 ACR [5, 6] and 2012 SLICC [3], have been designed to guide research and disease categorization.

Here we report a case of ANA-negative SLE that fulfilled 9/17 of SLICC criteria and 5/11 of ACR criteria, presenting as profound autoimmune neutropenia with strongly positive anti-neutrophil antibodies.

## 2. Case Presentation

The patient is a 53-year-old man of Hispanic descent with no significant past medical history who presented with fever and a rash over his face. At time of admission, he met SIRS criteria with CBC significant for pronounced neutropenia with pancytopenia, ANC <100, tachycardia, fever to 103, and diarrhea. Shortly following empiric infectious coverage with cefepime, vancomycin, and metronidazole, the patient's tachycardia and diarrhea resolved. However, he remained severely neutropenic with daily fever spikes and without other signs of infection.

**Figure 1:**
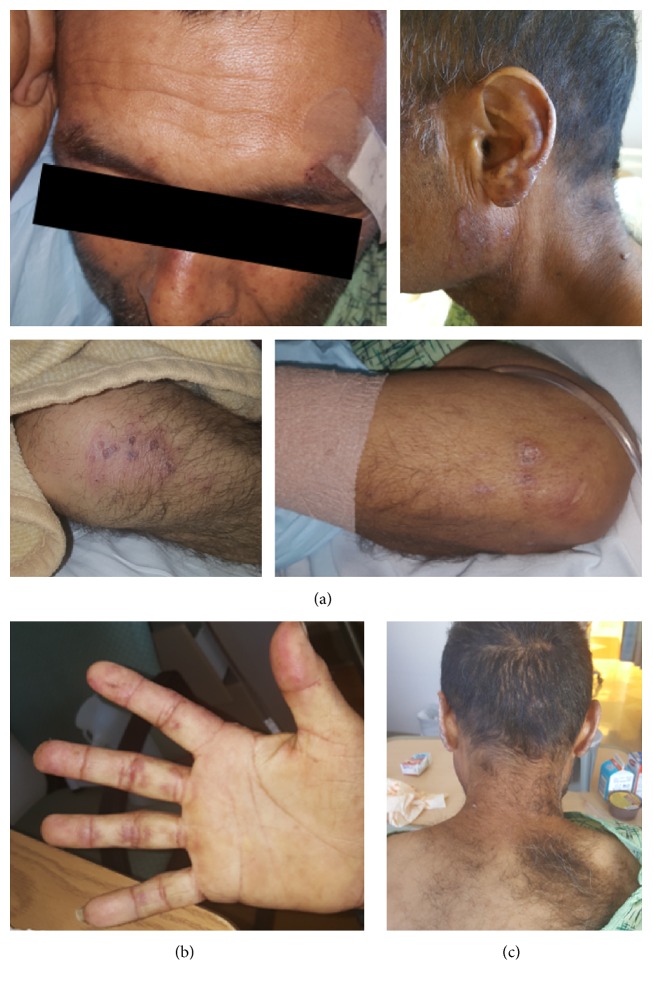
Physical findings of (a) violaceous patchy, discoid-like lesions above eyebrows, on side of face, ears, extensor surface of elbows, and knees, and malar-like rash on bridge of nose, (b) subcutaneous purpuric rash on palmar surface of fingers, and (c) alopecia of the head and hirsutism of the back.

Several interesting findings were present in the patient and shown in Figure 1, including painful mucocutaneous ulcers on upper and lower lips, possible malar rash across the bridge of his nose, discoid-like rash on various parts of his face, chest, extensor surface of his elbows, and possibly knees, and subcutaneous purpuric rash on the palmar surface of his fingers, as well as both alopecia and hirsutism with overgrowth of hair on his back. He denied any joint pain or photosensitivity. He reported having fever and diarrhea for a day, rash for a few weeks, and weight loss for the past several months. He had been homeless for the past year, with a history of heavy alcohol use and recent methamphetamine use for the past year, though he denied any IV drug use. He was recently brought into his son's home as he was steadily losing weight. There, he was noted to act strangely, seem confused, and continue to lose weight with poor appetite. He was brought into the hospital by his son as he developed a high fever with diarrhea. Initial lab results were remarkable for ANC <100, ferritin of 1237 ng/mL, positive direct Coombs' test, weakly positive anti-dsDNA, mildly elevated RF, and low C3/C4. See initial basic lab results in Table 1.

**Figure 2:**
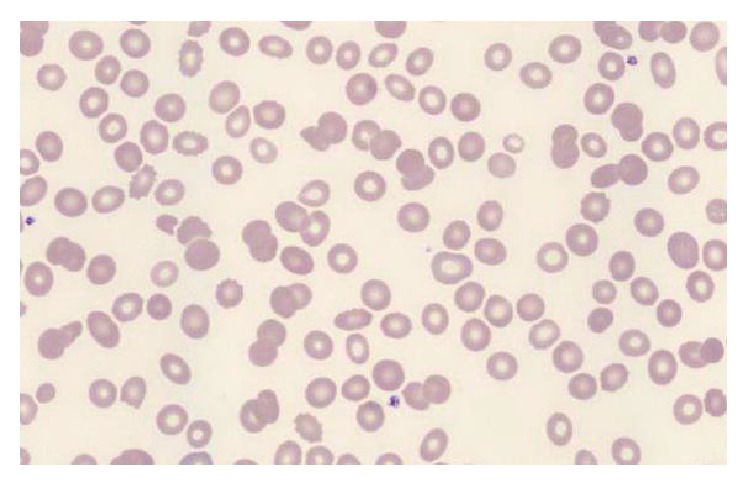
Peripheral blood smear showing normochromic RBCs with anisocytosis/poikilocytosis, thrombocytopenia, and paucity of PMNs and lymphocytes.

Throughout the next few days, an extensive workup was done to rule out any infectious causes or malignancy (see Table 2 for list of tests and results). All infectious lab tests returned negative except for a slightly positive galactomannan test with chest CT showing bilateral pleural effusions and a tree-in-bud appearance that resolved on a second chest CT. The patient remained asymptomatic though continued to spike daily fevers. Given this finding and the patient's cyclic fevers with profound neutropenia, empiric coverage with cefepime/meropenem and voriconazole was continued throughout hospitalization. To rule out malignancy, several peripheral smears and a bone marrow biopsy were examined, along with several lab assays for immunoglobulins and CD25. All findings were unremarkable. See Figure 2 for one example of patient's peripheral smear.

**Figure 3:**
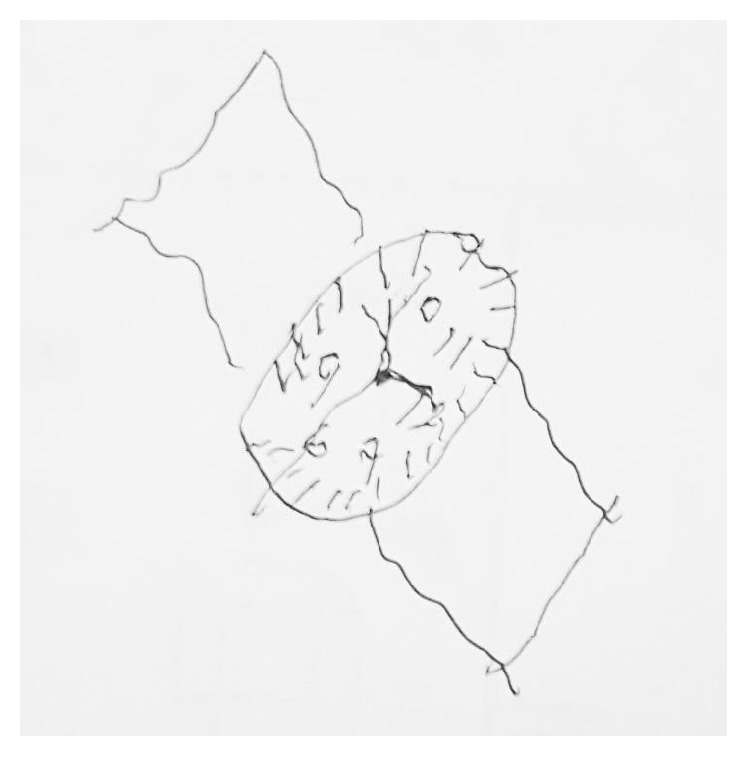
Patient's drawing of a watch at “ten past eleven,” part of MOCA test administered. Patient scored 6/30, with severe deficiencies in visuospatial/executive function, attention, and delayed recall.

Upon close daily interactions with our patient, we noted that he was tangential in his conversations with providers. He scored a 6/30 on MOCA, and we became concerned that there may be some neurological manifestations of his disease process. MRI of brain showed finding consistent with CNS vasculitis in the right motor cortex, hypothalamus, and mammillary bodies, suggestive of a systemic process such as SLE versus Korsakoff dementia secondary to heavy alcohol use. The patient's drawing on a clock on MOCA test can be seen in Figure 3.

Several days later, a skin biopsy of the violaceous, discoid-like lesion above the patient's eyebrows revealed atrophic dermatitis with marked telangiectasia, suggestive of lupus versus dermatomyositis.

Throughout hospitalization, we were hesitant to diagnose this patient with autoimmune disease as his rheumatologic lab tests were largely negative. However, when alternative infectious causes or malignancies were ruled out, we were more certain of an autoimmune etiology of disease. A comprehensive panel of rheumatologic markers was drawn, with results shown as follows.

*Panel of Rheumatologic/Immunologic/Autoimmune Lab Tests and Results*

*Lab Results*

Ferritin 1237 ng/mL
Iron 17 mcg/dL
TIBC 184 mcg/dL
CRP 2.0 mg/dL
ESR 40 mm/hr
ANA-negative
Anti-dsDNA 34 IU, low positive
ANCA negative
C3 31 mg/dL
C4 5 mg/dL
Cyclic citrulline peptide negative
Rheumatoid factor 66 IU/mL
DAT negative
ASO negative
Anti-MPO/RNP negative
Direct Coombs positive
Anti-cardiolipin negative
Anti-smith negative
Anti-SSA negative
Anti-SSB negative
Anti-histone negative
Serine protease negative
Cryoglobulin no precipitate

Based on several weakly positive results along with a high ferritin, we started patient on low dose steroid treatment with 20 mg IV solumedrol twice a day.

Shortly after beginning steroid treatment, the patient's daily fevers resolved, and his ANC started to improve, from ANC <100 to 900 a week after initiating therapy. Two and a half weeks after steroid therapy, his ANC improved to 2000. After discharge, it was found that he had high titers of anti-neutrophil antibodies via an assay performed by ARUP laboratories [7].

Though our patient was marker-negative for ANA, anti-Smith, anti-Ro, and anti-La, his lab results showed weakly positive anti-dsDNA, elevated ferritin to 1237 ng/mL, positive direct Coombs' test, mildly elevated RF, and low C3/C4. These findings, along with neutropenic fever that responded to steroid, pancytopenia, possible malar and discoid rash, CNS vasculitis, bilateral pleural effusions, and skin biopsy results, point to the high likelihood of SLE. Based on the findings above, the patient fulfilled 9/17 of SLICC criteria and 5/11 of ACR criteria for SLE classification.

## 3. Discussion

Our patient's most remarkable presentation of autoimmune neutropenia with cyclic fevers may be explained by a variety of etiologies, including viral, hematologic, or rheumatologic diseases [8]. Due to initial septic-like presentation, it was important to rule out any infectious or malignant causes of disease. Of note, we initially considered HIV with Kaposi sarcoma as our patient demonstrated severe neutropenia with the presence of violaceous rash on his face, chest, elbows, and knees. Other disease processes we considered included brucellosis, varicella, and herpes zoster (Table 2). Febrile neutropenia, commonly associated with cancer treatment, was reported in a patient with brucellosis and AML [9].

As all infectious and malignant work-ups were negative, we began to examine autoimmune and rheumatologic processes closely (see Panel of Rheumatologic/Immunologic/Autoimmune Lab Tests and Results). Initially, we were most surprised by the highly elevated ferritin level to 1237 ng/mL. Possible etiologies of elevated ferritin include HLH, MAS, RA, SLE, IBD, and other chronic diseases [10, 11]. Negative findings on bone marrow biopsy of hemophagocytosis and the lack of joint symptoms make the diagnosis of HLH, MAS, and RA less likely.

Other potential causes of profound neutropenia have been noted in literature, such as drug side effects of sulfonamides, metamizole, and clozapine [12]. Since 2003, levamisole, an antihelminthic with immunomodulatory properties, was found as a cocaine filler associated with neutropenia, ANCA positivity, and severe necrotic skin rash [13, 14]. In our patient, there exists the possibility of neutropenia secondary to cocaine ingestion due to a history of recent drug use. Though our patient denied IV drug use and endorsed methamphetamine use only, it is interesting to consider the possibility of levamisole-induced neutropenia, as the rash presentation was atypical for SLE or other rheumatologic/autoimmune diseases and the onset of disease was subacute within the span of a year.

Though autoimmune leukopenia is a common manifestation of SLE, the majority presents as lymphopenia, while moderate to severe autoimmune neutropenia only occurs in 5% of patients [1]. There are currently no standard guidelines for therapy. G-CSF and methylprednisolone have been tried with success in one study [15], while our patient demonstrated a robust response to low dose IV solumedrol. The mechanism of autoimmune neutropenia in SLE is unknown. However, possible mechanisms involve increased peripheral destruction, decreased bone marrow production, and increased margination of granulocytes [16]. Studies have noted an elevation in circulating IgG anti-neutrophil antibodies leading to complement activation [17] and an increase in TNF-related apoptosis inducing ligand (TRAIL) level [18, 19], as well as evidence for T cell- and monocyte-mediated suppression of granulocytopoiesis in the bone marrow of SLE patients [20].

Autoimmune neutropenia and its correlation with high anti-neutrophil antibody activity has previously been demonstrated [17]. In our patient, severe neutropenia correlated with a “strongly positive” anti-neutrophil antibody qualitative flow cytometry [7]. According to ARUP laboratories, this test may be positive in various autoimmune disorders, including Felty syndrome, SLE, and drug-induced neutropenia. A positive result is denoted as “weakly positive” when it is more than 2 standard deviations above the average value of a normal control population and “positive” when it is more than 3 standard deviations above. A positive test is not specific for any individual anti-neutrophil antibodies. As our patient was ANA-negative with a weakly positive anti-dsDNA, the diagnosis of SLE could remain controversial based on marker criteria alone. However, it is important to note that ANA has a sensitivity of 33.6%, while anti-dsDNA is only slightly better with a sensitivity of 57.1%, indicating that a significant portion of SLE cases may be missed based on these markers alone. Cases of marker-negative SLE have been reported in the past [21]. Recent studies have suggested that a positive ANA may disappear in some SLE patients overtime, with sensitivity dropping to 76% and positivity dropping from 98% to 71% in patients with established SLE [22, 23]. The finding of a strongly positive nonspecific anti-neutrophil antibody titer in our patient in the setting of marker negativity may indicate that more biomarkers are associated with SLE than those that are currently known. Investigation of these biomarkers may further elucidate the mechanism of autoimmune neutropenia.

Currently, the ACR and EULAR are working together to develop new classification criteria for SLE [24]. Using a four-phase process, this project seeks to review ANA sensitivity and specificity, weigh the importance of entry and additive criteria to eliminate redundancy and low-yield measures, and test the performance of these newly derived criteria against 1997 ACR and 2012 SLICC criteria. These new classification criteria take into account additional forms of cutaneous lupus and newly described autoantibodies, which may provide further guidance in clinical judgment of SLE. New promising biomarkers for SLE are currently under development, including microRNA [25], presence of urinary immune cells [26], and genetic biomarkers such as specific polymorphisms of MHC, interferon responsive factors, and integrins [27]. Together with updated classification criteria and new biomarkers, we are hopeful to see an increase in certainty of diagnosis for variable presentations of SLE.

## 4. Conclusion

Autoimmune leukopenia presents in 50–60% of patients with SLE, with the majority presenting as lymphopenia. Severe autoimmune neutropenia is an uncommon finding in less than 5% of patients and can correlate with high anti-neutrophil antibody activity despite a negative ANA. The mechanism of autoimmune neutropenia in SLE may involve a combination of increased peripheral destruction, decreased bone marrow production, and increased margination. As our patient had strongly positive anti-neutrophil antibodies in the setting of a negative ANA, it is possible that more biomarkers are associated with SLE than those that are currently known. Updated classification criteria and new biomarkers currently in development may greatly increase certainty of diagnosis for variable presentations of SLE.

## Figures and Tables

**Table 1:** Basic lab findings on presentation, most significant for elevated LDH and ANC <100.

Lab	Results
BMP	Sodium 132 mmol/L
	Potassium 3.9 mmol/L
	Chloride 97 mmol/L
	Bicarbonate 22 mmol/L
	Anion gap 13
	BUN 17 mg/dL
	Creatinine 1.00 mg/dL
	GFR >60 mL/min/1.73 m^2^
	Glucose 128 mg/dL
	Calcium 8.1 mmol/L

Liver panel	ALP 54 U/L
	ALT 87 U/L
	AST 152 U/L
	Direct bil 0.2 *μ*mol/L
	Total bil 0.55 *μ*mol/L
	Albumin 3.6 g/L
	Total protein 7.5 g/L

Blood chemistry	Phosphorous 2.2 mmol/L
	Magnesium 1.8 mEq/L
	LDH 710 U/L
	Lactate 1.4 mmol/L
	Uric acid 3.4 mmol/L
	Cortisol 49.6 mcg/dL

CBC	WBC 0.5 10^9^/L
	RBC 4.25 10^12^/L
	Hgb 10.5 g/dL
	Hct 31.6%
	MCV 74.4 fL
	MCH 24.7 fmol/cell
	MCHC 33.2 g/dL
	RDW 17.4%
	Plt count 122 10^9^/L
	MPV 10.1 fL
	Segs 6 10^9^/L
	Bands 2 10^9^/L
	Lymphocytes 61 10^9^/L
	Monocytes 29 10^9^/L
	ANC <100

Coagulation panel	PT 12.4 sec
	INR 1.1
	PTT 33.7 sec

**Table 2:** Panel of infectious lab tests and results. Mono may be falsely positive in autoimmune disease, confirmatory EBV test negative. Galactomannan positive.

Lab	Results
HIV rapid and RNA	Negative
HHV8	Negative
Parvovirus	Negative
Syphilis EIA, VDRL	Negative
CMV	Negative
Mono	Positive
EBV	Negative
Hepatitis A, B, C	Negative
GI pathogens	Negative
AFB	Negative
HSV	Negative
VZV	Negative
Aspergillus galactomannan	Positive
Histoplasma	Negative
*Coccidioides*	Negative
*Cryptococcus*	Negative
*Bartonella*	Negative
*Brucella*	Negative
*Coxiella*	Negative
